# 4314 The Impact of Axillary Surgery on Recurrence-Free Survival in Invasive Lobular Carcinoma (ILC) of the Breast

**DOI:** 10.1017/cts.2020.149

**Published:** 2020-07-29

**Authors:** Mary Kathryn Reed Abel, Jasmine Wong, Michael Alvarado, Cheryl Ewing, Laura J. Esserman, Catherine Park, Rita A. Mukhtar

**Affiliations:** 1 University of California, San Francisco

## Abstract

OBJECTIVES/GOALS: Clinical trials demonstrate that axillary lymph node dissection (ALND) is unnecessary for most breast cancer patients with 1-3 involved nodes, but whether this is true for those with ILC is unknown. We evaluate the impact of ALND on recurrence-free survival (RFS) in ILC and 1-3 positive nodes. METHODS/STUDY POPULATION: We performed a retrospective cross-sectional analysis of patients with ILC treated between 1992-2019 at our institution. All patients received either sentinel lymph node biopsy (SLNB) or ALND and underwent either breast conservation surgery (BCS) or mastectomy. The primary outcome was RFS, defined as the absence of locoregional or distant recurrence. RESULTS/ANTICIPATED RESULTS: Of 496 cases, 250 (50.4%) underwent BCS, and 246 (49.6%) underwent mastectomy. A total of 93% of patients were hormone receptor positive, and 89% had low or intermediate grade disease. Among patients with 1-3 positive nodes, there was no significant difference in 5- and 10-year RFS based on receipt of ALND for both BCS and mastectomy cohorts. Using a multivariate model, we found no association between ALND and RFS overall (HR = 0.98, 95% CI 0.36-2.7, p>0.20) and among those with 1-3 positive nodes (HR = 0.60, 95% CI 0.12-3.4, p>0.20). DISCUSSION/SIGNIFICANCE OF IMPACT: These findings support the safety of omitting ALND in patients with ILC and 1-3 positive nodes, regardless of whether they receive BCS or mastectomy. Further studies of axillary management in ILC, including imaging tools to predict nodal involvement and response to therapy, are warranted.

